# Undiagnosed synovial hemangioma of the knee: a case report

**DOI:** 10.1186/s13256-019-2107-7

**Published:** 2019-07-17

**Authors:** Yasuaki Tohma, Yoshio Mii, Yasuhito Tanaka

**Affiliations:** 1 Department of Joint Surgery, Kawasaki Saiwai Hospital, 31-27, Ohmiya-cho, Saiwai-ku, Kawasaki, Kanagawa Japan; 2 Department of Orthopedic Surgery, Nara Medical University, Nara, Japan; 3 Department of Orthopedic Surgery, National Hospital Organization Nara Medical Centre, Nara, Japan

**Keywords:** Synovial hemangioma, Knee joint, Intra-articular, Dysplasia of bone, Intermediate type

## Abstract

**Background:**

Synovial hemangioma of the knee is a rare benign tumor. Very rarely, the growth of bone is affected by long-term neglect of an intra-articular tumor. Our patient had not only various clinical symptoms but also dysplasia of the femoral bone. In this report, we aimed to raise awareness to prevent various disorders arising from an unnoticed or untreated hemangioma occurring within the knee joint.

**Case presentation:**

Our patient was a 41-year-old Japanese man who had had occasional discomfort in the right knee since elementary school. Although he had undergone radiography at several hospitals since childhood, no issues were reported; subsequently, he consulted our hospital. We performed magnetic resonance imaging and discovered a mass. The mass was homogeneous with low intensity on T1-weighted sequences and high intensity on T2-weighted sequences adjacent to the medial femoral condyle. The shape of the medial femoral condyle presented with a concavity in axial images, with irregular margins from the patellofemoral joint to the medial femoral condyle. Moreover, by using magnetic resonance angiography, we discovered a second mass. We decided to perform open surgery to achieve complete excision. Histological examination indicated a synovial hemangioma involving a cavernous hemangioma and irregular arteriovenous connections originating from the subsynovial tissue. The patient became asymptomatic after surgery, with no recurrence for more than 4 years.

**Conclusions:**

Synovial hemangioma is rare and difficult to diagnose in outpatient examinations because radiography has a limited diagnostic capacity. Magnetic resonance imaging and angiography are very useful. Nontreatment of intra-articular hemangiomas may lead to dysplasia of the bone and various clinical symptoms. Early complete excision may be instituted to reduce these risks of hemarthrosis.

## Background

Intra-articular hemangioma of the knee is a rare disease [1, 2]. We report a case of a patient with intra-articular and extra-articular hemangiomas of the knee deriving from the subsynovial tissue that had gone undiagnosed and untreated for 40 years. Long-term nontreatment of a tumor can cause dysplasia of the medial femoral condyle, as was observed in our patient. Diagnostic precautions and treatment methods have been reported to be beneficial in the assessment of this pathology. Furthermore, magnetic resonance imaging (MRI) and magnetic resonance angiography (MRA) are especially important in preoperative evaluations. In our patient, another tumor was discovered at the lateral side and was suspected to be a hemangioma. These tumors were present in the joint and outside the joint. This case represents an especially rare presentation of an intermediate-type hemangioma of the knee. Our aim is to report the diagnostic confirmation of intermediate-type hemangioma and the treatment.

## Case presentation

Our patient was a 41-year-old Japanese man who had had occasional discomfort in the right knee since elementary school. He had no pain at rest, but he experienced occasional pain when moving the knee. The pain had made it difficult for him to sit on his heels (Japanese *seiza* position) for the past year. Although he had undergone radiography and MRI examinations at several hospitals since childhood, no issues were reported; subsequently, he consulted our hospital.

At the time of his consultation, physical examination showed mild swelling and a loss of range of motion. The active range of motion of the knee was 30 degrees of extension and 120 degrees of flexion, improving to 10 degrees of extension when relaxed in a recumbent position. He had no instability of the knee. He had an intense fear of a sensation that something was caught in his knee joint. Blood test results were unremarkable. There was no tenderness at the femorotibial (FT) joint level. However, he complained of discomfort proximally, near the patellofemoral (PF) joint to the medial pouch. Careful palpation revealed an elastic soft tissue mass measuring about 3 cm. When his lower leg was hanging down while he was in a seated position, the blood vessels centered on this area became engorged.

**Fig. 1:**
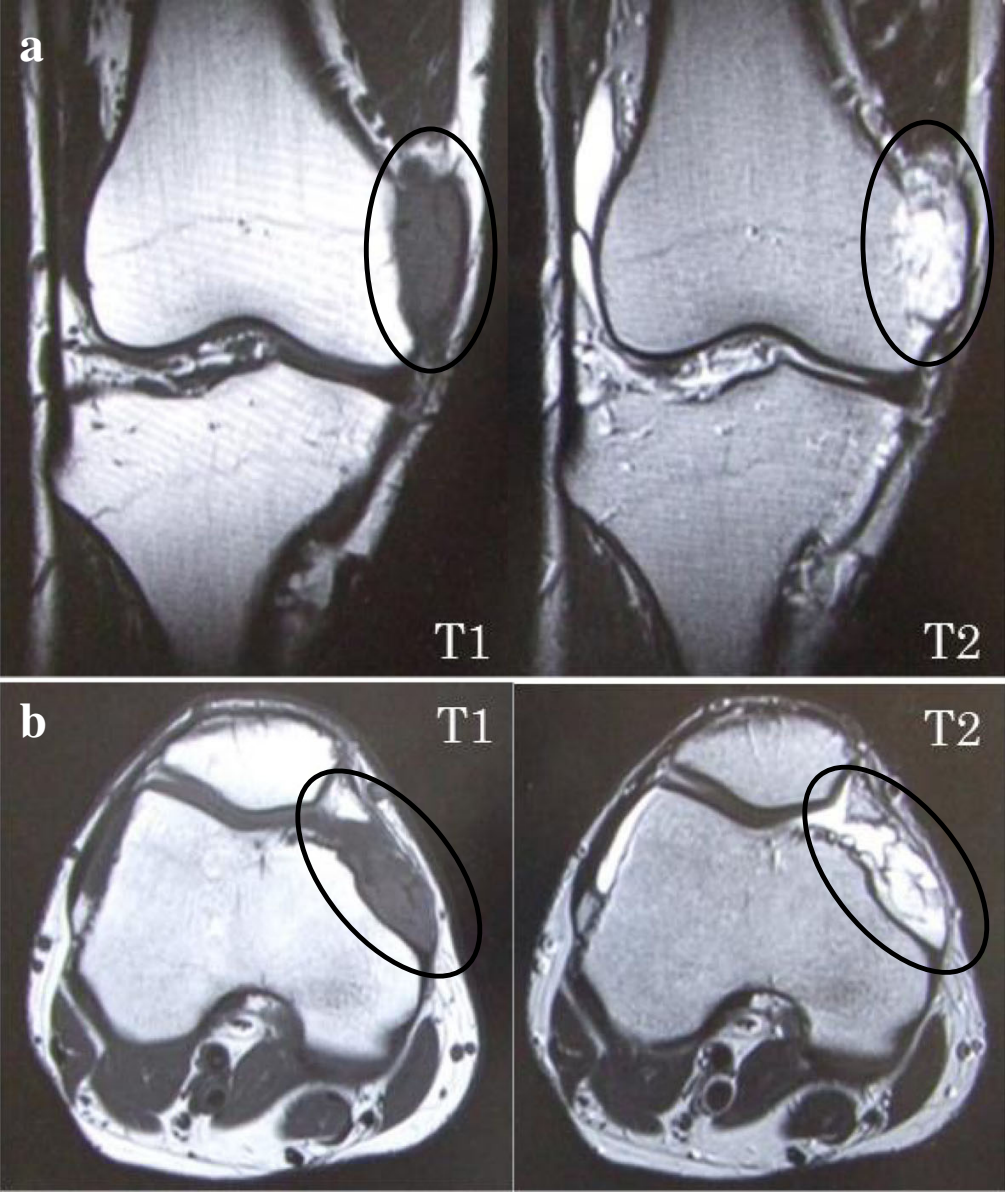
Magnetic resonance (MR) (**a**) coronal image and (**b**) axial image. MR images show a mass (circle). On axial T2-weighted MR images, the coronal view indicates an intra-articular mass from the patellofemoral to medial femorotibial joint level. Axial T1 image shows that the mass is homogeneous with low intensity. Axial T2 image shows that the mass is homogeneous with a high-intensity lesion. The high-intensity lesion is adjacent to the medial femoral condyle. The margin of bone is irregular. The ovals indicate the mass

**Fig. 2:**
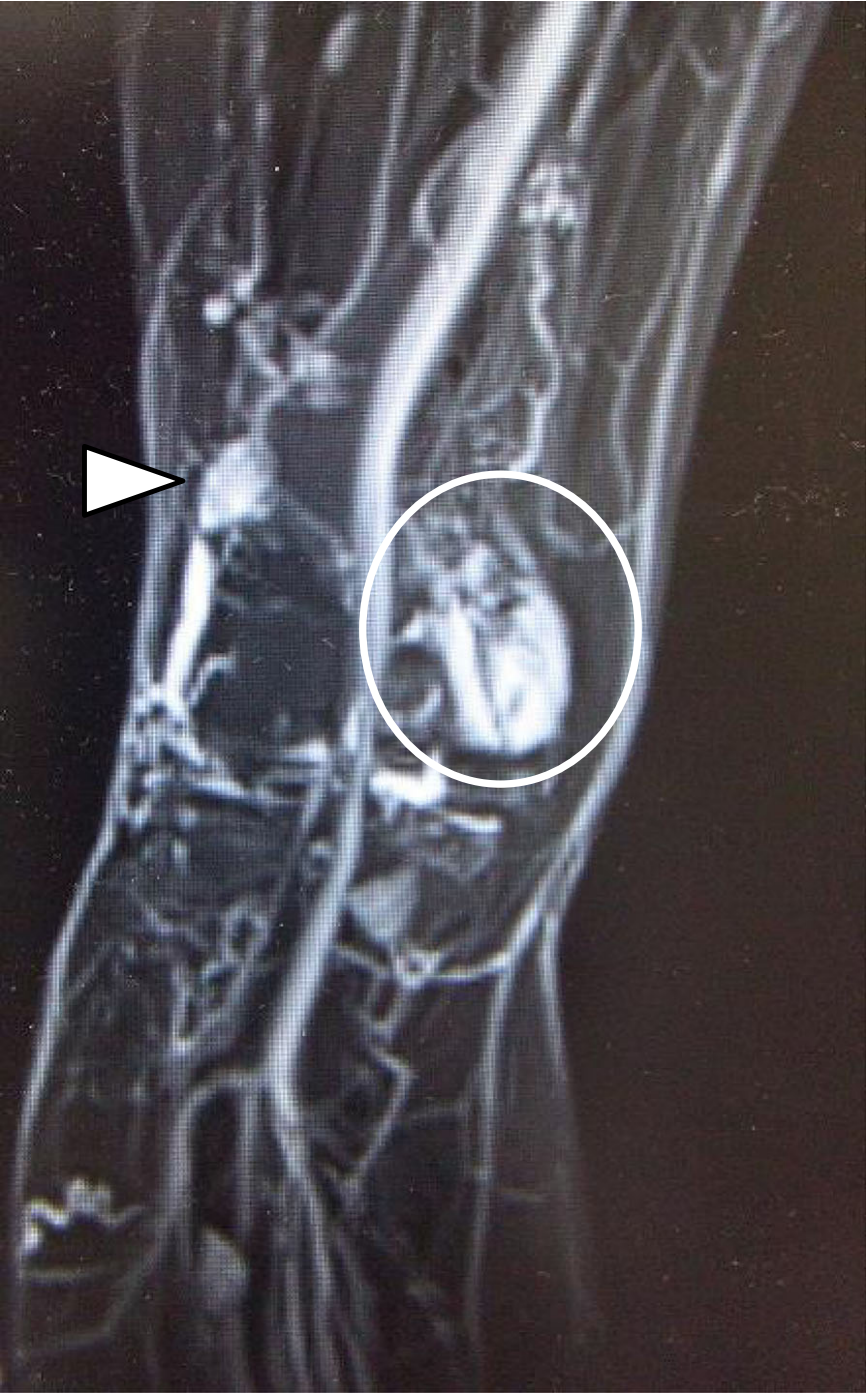
Magnetic resonance angiogram shows a hyperintense region (circle) on the medial side of the knee joint, consistent with the magnetic resonance image. Moreover, another hyperintense region is also indicated near the lateral femoral condyle (arrowhead) and was suspected of being a hemangioma

X-rays showed no obvious findings. MRI showed a mass measuring about 40 × 12 × 15 mm (Fig. 1a, b, ovals). The mass was homogeneous with a low intensity on T1-weighted sequences and high intensity on T2-weighted sequences adjacent to the medial femoral condyle. The shape of the medial femoral condyle presented with a concavity in axial images, with irregular margins from the PF to the medial femoral condyle. There were no abnormal signals in the intraosseous tissue. Suspecting a hemangioma, we performed MRA of the patient’s leg. The results showed a hyperintense region on the medial side of the knee joint, consistent with that seen on an MRI scan (Fig. 2, circle). Moreover, another hyperintense region measuring about 2 cm was also observed near the lateral femoral condyle (Fig. 2, arrowhead) and was suspected to be an extra-articular hemangioma. All evidence suggested intermediate-type hemangioma.

**Fig. 3:**
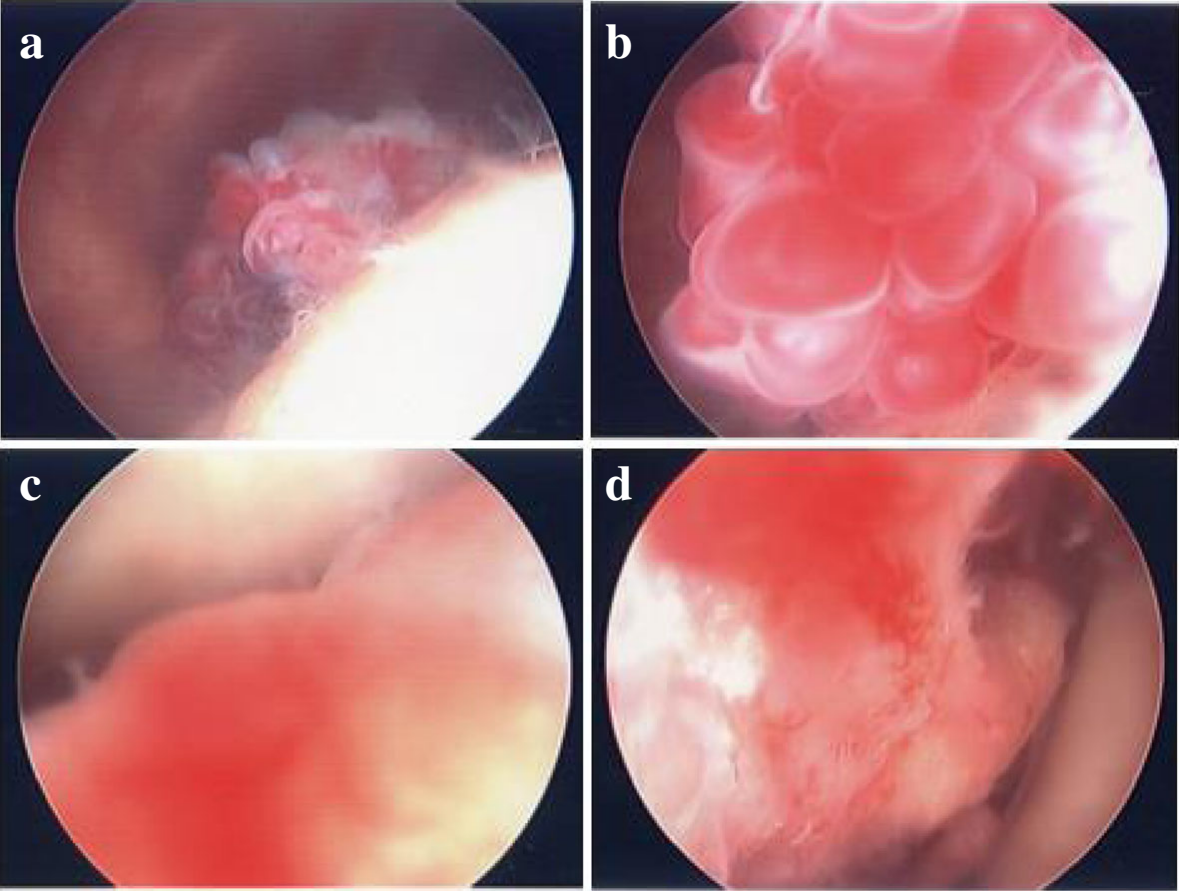
Arthroscopic views of the mass. **a** and **b** Synovial hemangioma of the lateral side. The mass has a multilocular tumor. This mass clearly consists of blood vessels. **c** and **d** Synovial hemangioma of the medial side. The mass spread from the patellofemoral joint to the medial femorotibial joint

**Fig. 4:**
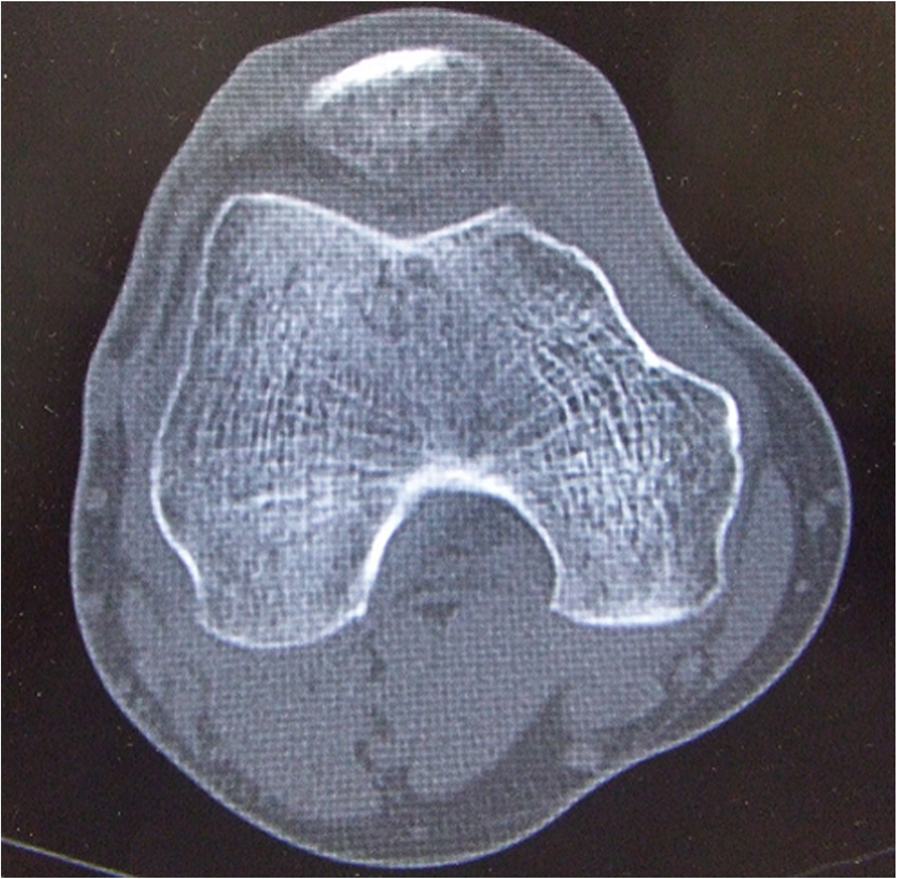
Axial computed tomographic image. The shape of the medial femoral condyle is partially concave

**Fig. 5:**
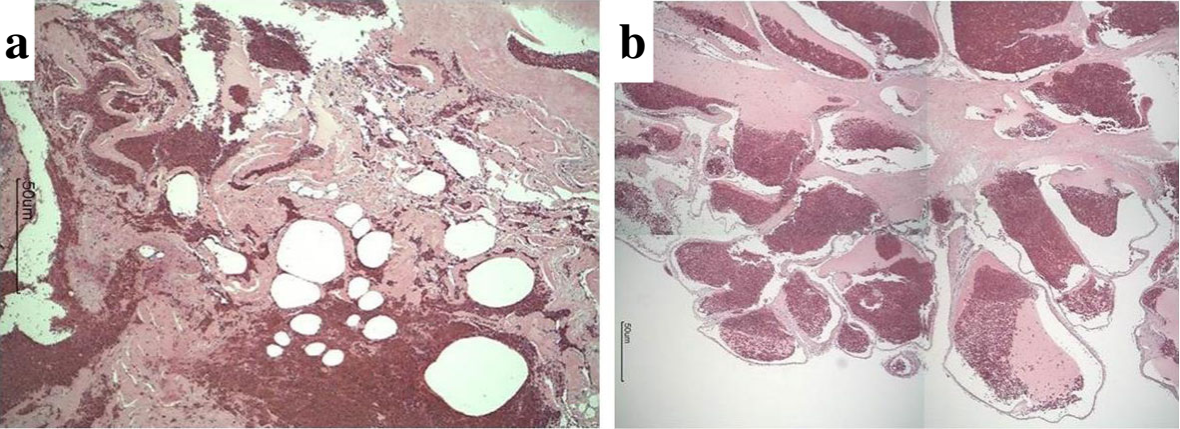
Histopathological examination revealed that the tumor was composed of numerous blood vessels. **a** The blood vessels are abnormally enlarged and connected to each other. **b** A cavernous hemangioma was found in synovial tissue (Hematoxylin and eosin (H&E) stain, original magnification × 40)

We performed surgery with the patient under general anesthesia. When his lower leg was hanging down on an operating table, remarkable engorgement of the blood vessels was observed. Arthroscopic examination was performed to confirm the nature, location, and extent of the mass (Fig. 3). The findings revealed a soft tissue mass that had grown from the PF to the medial pouch; the mass had spread from the medial epicondyle to the medial articular surface and was pinched by extension of the knee. The hyperintense region revealed by MRA was found at the lateral femoral condyle. There was no damage to the meniscus. A small incision to the lateral mass for subcutaneous access revealed a multilocular soft tissue mass measuring about 2 cm penetrating through the fascia of the vastus lateralis; the mass had the obvious appearance of a hemangioma. Because some of the engorged blood vessels were connected to the joint, we added an incision into the articular capsule. The mass infiltrated the synovial wall of the suprapatellar pouch; thus, the mass, measuring about 20 × 18 × 10 mm, was excised *en bloc*. On the basis of a report of synovial hemangioma occurring outside the knee joint [3], our patient’s case was considered to be a synovial hemangioma of the intermediate type originating from the intra-articular synovia, which was connected from the intra-articular region to the extra-articular region. With regard to the medial mass, because of its very large size, we decided to perform open surgery and visually identified the mass. The size of the mass was about 40 × 16 × 7 mm, and it spanned from the medial epicondyle to the medial articular surface. A part of the mass was attached to the cortex of the medial femoral condyle. We detached the mass from the bone surface and excised it. Upon resection, the medial condyle was observed to be dimpled with the shape of a crater (Fig. 4). The spread of the mass suggested a diffuse-type synovial hemangioma. Under direct observation, we performed complete hemostasis and sutured each layer. Histopathological diagnosis indicated a hemangioma originating from the synovial membrane or subsynovial connective tissue, as well as irregular arteriovenous connections (Fig. 5). Ultimately, the diagnosis of this patient was a diffused intermediate-type synovial hemangioma. The patient became asymptomatic after surgery, with no recurrence for more than 4 years.

## Discussion

Reports of hemangioma of the knee are rare; furthermore, diagnosis of hemangiomas is difficult and requires considerable time because of the general, nonspecific findings of hemarthrosis, which include localized pain or tenderness, swelling, and limited range of motion [1, 4–6]. Outside of intra-articular tumors, differential diagnosis includes pathologies such as meniscal injury, discoid meniscus, meniscal cyst, disorder of a medial patellar plicae, osteochondritis dissecans, juvenile idiopathic arthritis, and hemophilia. However, intra-articular hemangioma should be considered if there is an extensive history of arthralgia or nontraumatic hemarthrosis.

Painful or hemarthrotic intra-articular hemangioma of the knee is classified into the following two types: synovial hemangioma and arteriovenous malformation [7], which are also known as hemangiohamartomas. Both types arise from the synovial membrane and can cause nontraumatic hemarthrosis [7]. Moreover, they are the result of arthropathy caused by synovial hemorrhage, intra-articular hemorrhage, or repetitive mechanical stimulation. Both types are rare and thus difficult to diagnose and treat [8]. Bennett *et al.* have also divided synovial hemangiomas into diffuse and circumscribed types based on the manner of progression of the tumor [6]. Lewis *et al*. classified them as either a diffuse type or a localized type [3]. Finally, synovial hemangiomas are also classified by the site of occurrence (juxta-articular, intra-articular, or intermediate) [9]. The juxta-articular type often has joint swelling as its main symptom, whereas the intra-articular type is mainly characterized by intra-articular bleeding. Histological classification comprises venous, cavernous, capillary, arteriovenous, and sclerosing hemangioma types, with cavernous and capillary types being more common. Therefore, our patient was diagnosed with intermediate-type synovial hemangioma, with a histological classification of cavernous and arteriovenous hemangioma.

Regarding assessment, it has been reported that intra-articular masses determined to have noninfectious synovial proliferative processes exhibit specific imaging characteristics [10]. MRI examination is recommended as a tool for visualizing the size and characteristics of the lesion [2, 8, 11]. Using T1-weighted MRI, it is possible for changes in hemoglobin or hematoma over time to produce a relatively higher intensity of the lesion than that of water on the image [11]. Our patient also underwent MRA as a preoperative assessment of the vessels feeding the hemangioma and to provide a more complete picture of his condition. MRA makes it possible to refine the operative procedure and optimize measures to prevent bleeding. Moreover, another hemangioma was discovered at a different site in our patient, which had not been revealed during the medical examination. When hemangiomas have been present for an extensive period of time, more of them may also form at other sites. Thus, it is critical not to assume that only one mass is present in the case of a hemangioma. To identify all possible masses, we recommend performing MRA. Our patient also exhibited dysplasia of the femoral condyle. This dysplasia was inferred to be a secondary change caused by the mass since childhood; however, periosteal reaction and cortical destruction have also been reported as pathomechanisms, albeit at low rates (< 5%) [2]. Computed tomography should thus be performed as part of the preoperative examination to determine the presence of bone erosion or destruction. Some reports have described angioleiomyoma [12]; therefore, arthroscopy may also be advisable preoperatively.

Reported methods of treatment include embolization [13], radiotherapy [2], surgical excision (open surgery or arthroscopic surgery), and arthroscopic abrasion using a laser [14]. A wide excision is recommended to avoid hemangioma recurrence [15]. Treatment by arthroscopy alone should be limited to cases with small and localized tumors, when the lesion is properly assessed by diagnostic imaging and determined to be treated arthroscopically. An invasive, complete resection should be chosen in cases of multifocal, extensive tumors, such as that in our patient. Early treatment is also preferable because synovial hemangiomas result in arthropathy due to repetitive intra-articular hematomas and infiltration of the muscular layer, fat layer, and cortical bone [2]. Our patient also presented with bone dysplasia due to long-term nontreatment of the condition. Delayed treatment reportedly results in osteoporosis, advanced maturation of the epiphyses, a discrepancy in leg length, and arthropathy simulating hemophilia [2]. In our patient, approximately 40 years had elapsed before diagnosis of the condition; with an earlier diagnosis, the osseous changes might have been avoided.

## Conclusion

Synovial hemangioma of the knee is rare and difficult to diagnose in outpatient examinations because radiography has a limited diagnostic capacity. If the swelling persists, a joint puncture should be performed, and the joint fluid should be evaluated. If blood is found, hemangioma should be suspected. In that case, both MRI and MRA are useful and should be performed. Nontreatment of intra-articular hemangioma may lead to dysplasia of the bone and various clinical symptoms such as loss of range of motion, catching, and arthropathy. Early complete excision may be instituted to reduce these risks of hemarthrosis.

## References

[1] Bouchut E. Tumeur erecite de l’articulation du genou. Gaz Hop (Paris). 1856;29:379–81.

[2] Choudhari P, Ajmera A. Haemangioma of knee joint: a case report. Malays Orthop J. 2014;8(2):43–5. 10.5704/MOJ.1407.002 PMC4181078 25279092

[3] Lewis RC Jr, Coventry MB, Soule EH. Hemangioma of the synovial membrane. J Bone Joint Surg Am. 1959;41-A(2):264–71. 13630962

[4] Devaney K, Vinh TN, Sweet DE. Synovial hemangioma: a report of 20 cases with differential diagnostic considerations. Hum Pathol. 1993;24(7):737–45. 10.1016/0046-8177(93)90010-e 8319952

[5] Halborg A, Hansen H, Sneppen HO. Haemangioma of the knee joint. Acta Orthop Scand. 1968;39(2):209–16. 10.3109/17453676808989454 5702198

[6] Bennett GE, Cobey MC. Hemangioma of joints: report of five cases. Arch Surg. 1939;38:487–500.

[7] Akgün I, Kesmezacar H, Oğüt T, Dervişoğlu S. Intra-articular hemangioma of the knee. Arthroscopy. 2003;19(3):E17. 10.1053/jars.2003.50068 12627134

[8] Yercan HS, Okcu G, Erkan S. Synovial hemangiohamartomas of the knee joint. Arch Orthop Trauma Surg. 2007;127(4):281–5. 10.1007/s00402-006-0140-7 16609865

[9] Jacobs JE, Lee FW. Hemangioma of the knee joint. J Bone Joint Surg Am. 1949;31(4):831–6. 18142925

[10] Sheldon PJ, Forrester DM, Learch TJ. Imaging of intraarticular masses. Radiographics. 2005;25(1):105–19. 10.1148/rg.251045050 15653590

[11] Cotten A, Flipo RM, Herbaux B, Gougeon F, Lecomte-Houcke M, Chastanet P. Synovial haemangioma of the knee: a frequently misdiagnosed lesion. Skelet Radiol. 1995;24:257–61. 10.1007/BF00198409 7644936

[12] Okahashi K, Sugimoto K, Iwai M, Oshima M, Takakura Y. Intra-articular angioleiomyoma of the knee: a case report. Knee. 2006;13(4):330–2. 10.1016/j.knee.2006.03.002 16647261

[13] Gould ES, Potter HG, Huvos A, Furie R, Crystal KS. Case report 671: arteriovenous malformation of the right lower extremity with associated intraosseous hemangiomatosis. Skeletal Radiol. 1991;20(4):303–5. 10.1007/BF02341672 1853226

[14] Shapiro GS, Fanton GS. Intraarticular hemangioma of the knee. Arthroscopy. 1993;9(4):464–6. 10.1016/s0749-8063(05)80323-8 8216580

[15] Bruns J, Eggers-Stroeder G, von Torklus D. Synovial hemangioma—a rare benign synovial tumor: report of four cases. Knee Surg Sports Traumatol Arthrosc. 1994;2(3):186–9. 10.1007/BF01467924 7584204

